# Characterization of lignin-degrading enzymes (LDEs) from a dimorphic novel fungus and identification of products of enzymatic breakdown of lignin

**DOI:** 10.1007/s13205-016-0384-z

**Published:** 2016-02-13

**Authors:** Lipin Dev Mundur Sahadevan, Chandra Shekhar Misra, V. Thankamani

**Affiliations:** 1 School of Bio Sciences and Technology, VIT University, Vellore, 632014 Tamil Nadu India; 2 Department of Biotechnology, Prathyusha Institute of Technology and Management, Tiruvallur, 602025 Tamil Nadu India; 3 Avenida Rovisco Pais, 1, 1049-001 Lisbon, Portugal

**Keywords:** Laccase, Lignin, Lignin peroxidase, Manganese peroxidase, MVI.2011, Biofuels

## Abstract

Lignin is a major component of all plants, the degradation of which remains a major challenge to date owing to its recalcitrant nature. Several classes of fungi have been studied to carry out this process to some extent, but overall the process remains inefficient. We have isolated a novel alkalophilic dimorphic lignin-degrading Deuteromycete from soil, identified as “uncultured” and coded as MVI.2011. Supernatant from 12-h culture of MVI.2011 in optimized mineral medium containing lignin pH 9.0 was analysed for Lignin Peroxidase, Manganese Peroxidase and Laccase. Enzyme purification was carried out by standard protocols using ammonium sulphate precipitation followed by further purification by Gel Permeation Chromatography. Analysis of total protein, specific enzyme activity and molecular weight of the GPC-purified LiP, MnP and Laccase showed 93.83 μg/ml, 5.27 U/mg, 42 kDa; 78.13 μg/ml, 13.18 U/mg, 45 kDa and 85.81 μg/ml, 4.77 U/mg, 62 kDa, respectively. The purified enzymes possessed high activity over a wide range of pH (4–11), and temperature (30–55 °C). The optimum substrate concentration was 20 μg/ml of lignin for all the three enzymes. CD spectra suggested that the predominant secondary structure was helix in LiP, and, turns in MnP and Laccase. The breakdown products of lignin degradation by MVI.2011 and the three purified enzymes were detected and identified by FTIR and GC–MS. They were oxalic acid, hentriacontane, derivatives of octadecane, nonane, etc. These vital compounds are certain to find application as biofuels, an alternate energy source in various industries.

**Electronic supplementary material:**

The online version of this article (doi:10.1007/s13205-016-0384-z) contains supplementary material, which is available to authorized users.

## Introduction

Fungi are well-known microorganisms found to degrade lignin and have been extensively studied (Evans and Hedger 2001; Hatakka 2001). White rot fungi are believed to be the only and most effective degraders of lignin which comprise of several hundred species of Basidiomycetes and a few species of Ascomycetes. They are capable of degrading lignin component of wood completely to CO_2_ and water. Of late, more diverse and taxonomically distinct fungi have been studied for their lignin-degrading capacity and brought under the purview of useful application in effluent treatment from various pulp and paper industries (Sahadevan et al. 2013). White rot fungi contain specific enzymes Lignin Peroxidase (LiP), Manganese Peroxidase (MnP), Laccase, and hydrogen peroxide-generating enzymes. Along with these enzymes, ROSs (Reactive oxygen species) is also considered to be an important agent for wood decay by fungi. Various combinations of these enzymes are produced by different fungi which suggest different mechanisms of lignin degradation (Sahadevan et al. 2013).

So far ligninolytic enzymes have been known to be produced by various fungi including *T. versicolor*, *Phlebia fascicularia*, *Dichomitus squalens*, *Daedalea flavida*, *Phlebia floridensis and P. Radiate* (Arora et al. 2002), but most of them have failed to bring about efficient degradation of lignin at the high pH of pulp paper and such other industrial effluents. Literature survey shows that in general, fungi require a minimum of 2–4 weeks for growth and production of extracellular lignin-degrading enzymes. They also prefer lower pH (4–7) and temperature for lignin decomposition activity (Yang et al. 2011). No single organism has been reported to produce all three enzymes, and, in equal proportions (Li et al. 2001).

MVI.2011, a novel dimorphic alkalophilic fast-growing Deuteromycete was isolated and reported to degrade lignin, melanoidin and dyes (Thankamani et al. 2002; Dev and Thankamani 2012, 2013). The strain isolated and identified resembles *Mycelia sterilia* in several of its morphological properties and functional characterization; however, further study had to be carried to ascertain its exact species. The unique properties of strain MVI.2011 secreting a variety of enzymes, long shelf life at ambient temperatures, non-sporing nature, rapid growth, and highly alkalophilic nature open up a huge potential for large-scale application of the fungus to treat industrial effluents from pulp paper, distillery, and dye industry. The present study describes the purification and characterization of lignin-degrading enzymes, LiP, MnP and Laccase from MVI.2011 to provide a suitable biological alternative to treat highly alkaline effluent such as pulp and paper industry waste water in particular. We also identified key products obtained by the degradation of lignin by MVI.2011 and three purified enzymes (LiP, MnP and Laccase) which could have potential application as alternate energy source in industries like food processing, biofuels, etc.

## Materials and methods

### Organism

MVI.2011, an alkalophilic fungus reported to degrade melanoidin and remove colour from molasses-based distillery spent wash maintained on SDA slopes at 4 °C, was used for this work (Thankamani et al. 2002).

### Flask setup

A pure single colony of MVI.2011 was inoculated into 250 ml of the previously optimized modified mineral medium (mineral salts—0.01 % each of sodium phosphate, potassium phosphate and magnesium sulphate, 0.5 % peptone, 1 % glucose and 0.1 % of lignin, pH 9.0) and incubated at ambient temperature for 12 h. The broth was centrifuged at 6000 rpm for 20 min at 4 °C. The culture supernatant was used for further characterization viz., estimation of total proteins, enzyme assays, concentration and fractionation by ammonium sulphate precipitation and purification by gel permeation chromatography.

### Assays

#### Protein estimation

Total soluble protein in each fraction was estimated by Lowry’s method (Lowry et al. 1951).

#### Ammonium sulphate precipitation

The entire purification steps and concentration of proteins were performed at 4 °C. Small pre-weighed quantities of ammonium sulphate were added to 250 ml of culture supernatant from 20 to 80 % saturation. Each precipitated fraction was separated by centrifugation at 10,000 rpm for 15 min at 4 °C, dissolved in minimum volume of 0.1 M Tris–HCl (pH 9.0) and dialyzed twice for 6–8 h against the same buffer (Adinarayana et al. 2003). The dissolved fractions were stored at 4 °C.

### Enzyme assays on culture filtrate and precipitates

The ligninolytic enzymes’ activity (LiP, MnP and Laccase) were estimated in the crude culture filtrate and ammonium sulphate precipitates by standard protocols described below. The precipitates that showed the highest activity for the above enzymes were subjected to gel permeation chromatography.

#### Lignin peroxidase (LiP)

LiP was determined in a 12-h culture supernatant referred to as crude enzyme. The reaction mixture contained Azur B (32 μM), sodium tartrate buffer (50 mM, pH 3), and 0.5 ml of crude enzyme (10 μg of each of the ammonium sulphate precipitate fractions). The reaction was initiated by adding 0.5 ml of H_2_O_2_. One unit of enzyme activity was equivalent to an absorbance decrease at 310 nm of 0.1 unit min^−1^ ml^−1^ (Archibald 1992; Arora and Gill 2001).

#### Manganese peroxidase (MnP)

MnP assay was performed on the basis of oxidation of phenol red. Reaction mixture contained 1 ml of sodium succinate buffer (50 mM, pH 4.5), 1 ml sodium lactate (50 mM, pH 5), 0.4 ml manganese sulphate (0.1 mM), 0.7 ml phenol red (0.1 mM), 0.4 ml H_2_O_2_ (50 μM), gelatine 1 mg ml^−1^ and 0.5 ml of crude enzyme (10 μg of each of the ammonium sulphate precipitate fractions). The reaction was initiated by adding H_2_O_2_ and incubated at 30 °C. 40 μl of 5 N NaOH was added to 1 ml of the reaction mixture. Absorbance was measured at 610 nm. After every minute the same steps were repeated with 1 ml of the reaction mixture up to 4 min. One unit of the enzyme activity was equivalent to an absorbance increase of 0.1 unit min^−1^ ml^−1^ (Orth et al. 1993).

#### Laccase

Laccase activity was measured using Guaiacol as the substrate. The reaction mixture containing 3.8 ml acetate buffer (10 mM, pH 5), 1 ml of Guaiacol (2 mM) and 0.2 ml of the enzyme extract (10 μg of each of the ammonium sulphate precipitate fractions) was incubated at 25 °C for 2 h. The absorbance was read at 450 nm. Laccase activity was expressed as colorimetric unit ml^−1^ (CU ml^−1^) (Arora and Sandhu 1985).

### Gel permeation chromatography

The ammonium sulphate precipitate fractions showing highest LiP, MnP or laccase enzyme activities were injected into Sephadex G-75 column (2 × 20 cm) equilibrated with 0.1 M Tris–HCl (pH 9.0). Before loading the sample, the column was washed with 250 ml of the same buffer. 2 ml fractions were collected at a flow rate of 40 ml h^−1^. Both protein and enzyme activities were estimated in each fraction.

In column chromatography samples, the concentration of protein was estimated by measuring their absorbance at 280 nm. The fractions showing presence of protein and the particular enzyme activity were pooled together, lyophilized and dissolved in 0.1 M Tris–HCl (pH 9.0) for further analysis (Moreira et al. 2003).

### HPLC

The fractions obtained from GPC with respective enzyme (LiP, MnP or Laccase) activity were analysed by HPLC (sample—20 µl; Mobile phase—Methanol:Water 80:20 (v/v); flow rate—1 ml/min; stationary phase column—wakosil C-18; Analysis of resultant peak-UV-detector) for purity.

### SDS–poly acrylamide gel electrophoresis

Crude culture filtrate, ammonium sulphate precipitates showing enzyme activity and GPC-purified enzyme fractions were subjected to SDS-PAGE according to Laemmli (1970) using 12 % polyacrylamide gel. Protein bands were detected by Coomassie brilliant blue (0.1 %) staining. Standard molecular weight markers from 11 to 65 kDa were also loaded.

### Conformation of purified enzyme proteins

Circular dichroism (CD) spectra of purified LiP, MnP and Laccase enzymes obtained from GPC were analysed by JASCO J-715 CD spectrophotometer. The conformational changes of the secondary structure of each purified enzyme in the presence of the substrate (lignin) were also analysed. 5 µg of each purified enzyme was dissolved in 5 ml of 0.01 M of Tris buffer (pH 9.0) and was used for the analysis. 2 µl of H_2_O_2_ was added as initiator for enzyme substrate reaction for both LiP and MnP activity. The secondary structure was obtained using the software JASCO Corp., J-715 manufacturer (Tokyo, Japan). CD spectrum of pure lignin was used as a negative control.

### Characterization of purified LiP, MnP and Laccase enzymes

For all tests, except influence of concentration of substrate, 10 µg each of purified LiP, MnP and Laccase enzyme were mixed with 20 µg/ml of ligno-sulphonate substrate.

### Influence of pH

The pH of purified LiP, MnP and Laccase enzyme–substrate reaction mixtures were adjusted to pH 4, 5, 6, 7, 8, 9, 10 and 11 with suitable buffers such as 0.1 M citrate, phosphate and Tris–HCl and incubated at ambient temperature (25–30 °C). After 1 h, aliquots from each set were tested and lignin breakdown was estimated by performing enzymes assays as described earlier.

### Influence of temperature

Influence of temperature was studied at media pH 9.0. The purified enzyme substrate mixtures in seven separate tubes and control were incubated in a water bath at 22, 26 30, 37, 40, 45, 50, 55 and 60 °C for 1 h and then tested for reduction in colour by performing the enzyme assays as described earlier.

### Influence of substrate concentration

10 µg of the purified enzymes was incubated with each of the substrate (lignin) concentrations ranging from 5 to 30 µg/ml in 0.1 M Tris buffer at ambient temperature (25–30 °C) for 1 h at pH 9. The buffer without substrate was taken as control. Lignin breakdown was measured by reduction in colour by performing enzyme assays as described above.

### Influence of incubation time

The enzyme and substrate (lignin) mixtures were incubated at ambient temperature for various durations starting from 0, 15, 30, 60, 90 and 120 min, respectively. Lignin breakdown by the three enzymes was measured by reduction in colour as described above.

### Identification of lignin breakdown compounds

The metabolic breakdown of lignin by MVI.2011 in broth, and, by MVI.2011 purified enzymes viz. LiP, MnP and Laccase was analysed using GC–MS. Tubes containing minimal mineral media with 0.1 % lignin were inoculated with MVI.2011 and incubated for 12 h at ambient temperature. The uninoculated broth was used as control. The culture was centrifuged at 12,000 rpm for 20 min. For products of enzymatic breakdown purified enzyme–substrate mixtures at optimized proportion were used. The supernatant was used for Fourier-transform infrared spectroscopy (FTIR) and Gas chromatography–mass spectrometry (GC–MS) analysis. For GC–MS, the supernatant was extracted by solvent–solvent extraction using double the volume of ethyl acetate. Direct supernatant was used for FTIR analysis.

#### Fourier-transform infrared spectroscopy

Lignin was broken down by MVI.2011 in culture, and, also using the three purified enzymes viz. Lignin Peroxidase, Manganese Peroxidase and Laccase and analysed using FTIR spectrophotometer (Shimadzu, Resolution: 4 cm^−1^). Samples were analysed in Technology Business Incubator, VIT University. For the FTIR study, approximately 10 µl each of MVI.2011 was inoculated into 100 µl of optimised medium containing 0.1 % lignin, incubated at optimized conditions, centrifuged (10,000 rpm, 4 °C) and culture supernatant preserved in the refrigerator for further studies. GPC-purified samples of LiP, MnP and Laccase produced by MVI.2011 were mixed with substrate lignin (ligno-sulphonate) at optimized ratio prepared in Tris–HCl buffer, pH 9.0 and incubated for 1 h at ambient temperature (30–32 °C). Medium control, culture supernatant, lignin enzyme mixtures were all preserved in the refrigerator and analysed. For FTIR study, approximately 10 µl each of the above samples were encapsulated in 40 mg of KBr pellet (Sigma, USA) to prepare translucent sample disks. The FTIR spectra of all samples were recorded.

#### Gas chromatography–mass spectrometry

The breakdown compounds were analysed by gas chromatography–mass spectrometry (GC–MS) (Perkin Elmer- Clarus 680). The parameters specific to the GC–MS are described below:

Oven: Initial temp 60 °C for 2 min initially at 10 °C/min rose to 300 °C, hold 4 min; total run time: 30.00 min; volume = 1 μL; flow rate: 1 mL/min; carrier gas = helium. Mass condition (Ei)–solvent delay = 2.00 min; transfer temp = 230 °C; source temp = 230 °C; scan: 50–600 Da.

The identification of degradation product was performed by comparing the mass spectra of the degradation product with that of the National Institute of Standards and Technology (NIST) library available in the instrument and also by comparing the retention time (RT) with those of available authentic compounds.

## Results and discussion

### Purification of LiP, MnP and Laccase enzymes

#### Culture filtrate (crude enzyme)

**Fig. 1:**
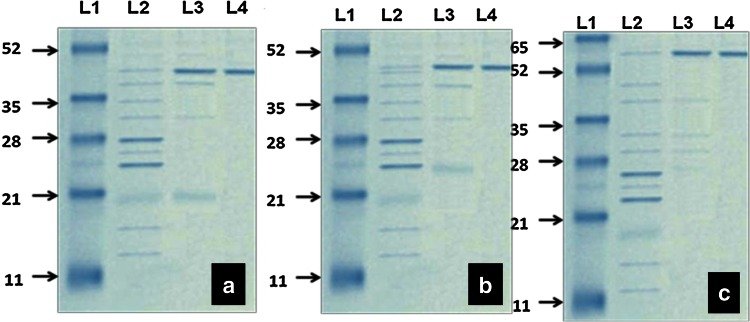
SDS PAGE of purified enzymes **a** protein marker (*Lane-L1*), crude enzyme (*Lane-L2*), ammonium-precipitated fractions (*Lane-L3*), purified LiP (*Lane-L4*); **b** protein marker (*Lane-L1*), crude enzyme (*Lane-L2*), ammonium-precipitated fractions (*Lane-L3*), purified MnP (*Lane-L4*); **c** protein marker (*Lane-L1*), crude enzyme (*Lane-L2*), ammonium-precipitated fractions (*Lane-L3*), purified Laccase (*Lane-L4*)

The protein content of the crude enzyme was found to be 688.3 µg/ml and specific activities of the LiP, MnP and Laccase enzymes were 0.1380, 0.290 and 0.101 U/mg, respectively (Table 1). In SDS-PAGE the crude enzyme showed the presence of 12 bands and two of the bands were prominent. The molecular weight of the bands ranged from approximately 15 to 52 kDa (Fig. 1).

**Table 1:** Purification summary for lignin-degrading enzymes (LDEs) produced by novel fungus MVI.2011

Purification steps	Protein (µg/ml)			Activity (U/ml)			Specific activity (U/mg)			Protein recovery (%)			Purification fold		
	LiP	MnP	Laccase	LiP	MnP	Laccase	LiP	MnP	Laccase	LiP	MnP	Laccase	LiP	MnP	Laccase
Crude extract	688.37	688.37	688.37	0.095	0.2	0.07	0.1380	0.290	0.101	100	100	100	–	–	–
Ammonium sulphate precipitation	161.27	120.23	113.44	0.2	0.86	0.32	1.240	7.15	2.82	23.43	17.46	16.47	8.98	24.65	27.92
Sephadex G-75 gel filtration	93.83	78.13	85.81	0.495	1.03	0.41	5.27	13.18	4.77	13.63	11.35	12.46	38.18	45.44	47.30

*LiP* lignin peroxidase, *MnP* manganese peroxidase

#### Analysis of ammonium sulphate precipitate fractions

Ammonium sulphate precipitation yielded seven fractions between 20 and 80 % saturation. The fractions were labelled as F1 (20 %), F2 (30 %), F3 (40 %), F4 (50 %), F5 (60 %), F6 (70 %) and F7 (80 %), respectively. The protein concentration of the fractions were F1—48.13, F2—161.27, F3—120.23, F4—113.48, F5—147.44, F6—189.06 and F7—135.58 µg/ml (Data not shown). Enzyme activity assay of the fractions showed that LiP, MnP and Laccase resided in F2, F4 and F5, respectively (Fig. 1). There were five faint bands and one prominent band in both F2 and F4 and five bands in F5 with molecular weights 25–45, 20–52 and 27–62 kDa, respectively. The specific activities for LiP, MnP and Laccase were 1.240, 7.5 and 2.82 U/mg, respectively (Fig. 1; Table 1).

#### Gel permeation chromatography

In GPC, the ammonium sulphate fraction F2 showed a single peak of 93.83 µg/ml of protein with only LiP activity (specific activity 5.27 U/mg). While fraction F3 showed a peak with 78.13 µg/ml of protein with only MnP activity (specific activity 13.18 U/mg) fraction F4 contained a peak with 85.81 µg/ml of protein and showed Laccase activity (specific activity 4.77 U/mg) (Data not shown). The purified LiP, MnP and Laccase fractions on SDS-PAGE formed single bands with molecular weights 42, 45 and 62 kDa, respectively (Fig. 1; Table 1).

### HPLC analysis

GPC-purified fractions showing LiP, MnP and Laccase activities produced peaks with retention time 3.493, 2.317, 2.872 and heights 41,681, 315,559, 98,187, respectively (Online resource 1a–c).

#### Purification

All the three enzymes studied could be purified to homogeneity. The specific activity, protein recovery and purification fold are shown in the Table 1.

In a similar study by Saito et al. (2003), the Laccase enzyme was purified 2.9-fold with an overall yield of 26.5 %. Dedeyan et al. (2000) could achieve purification of Laccase obtaining 19 mg of purified enzyme with specific activity of 13U/mg corresponding to the final yield of 31 %. There are reports that LiP and alcohol oxidase are highly expressed during lignin degradation (Sato et al. 2009). A comparison of MVI.2011 with other reports show that this isolate produced ten times higher levels of LiP (9.39 Uml^−1^) within 18 h (Arora et al. 2002; Dev and Thankamani 2012).

It has been reported that *P. chrysosporium* produces high LiP and MnP activity but no Laccase activity (Kerem et al. 1992; Arora 1995) and it has been confirmed in studies using model lignin compounds (Ruttimann et al. 1992; Hammel et al. 1994). The absence of Laccase in *P. chrysosporium* might be responsible for lesser lignin utilization when compared to *Phlebia sp*. as reported (Kirk and Farrel 1987; Kerem et al. 1992; Hatakka 1994; Thurston 1994). But there is a contrasting report by Srinivasan et al. (1995) who showed Laccase production by *P. chrysosporium* in defined medium containing cellulose. Arora et al. (2002) studied ligninolytic enzymes of T*. versicolor*, *Phlebia fascicularia*, *Dichomitus squalens*, *Daedalea flavida*, *Phlebia floridensis* and *P. Radiate* ^4^. They have reported that *Phlebia* spp. was capable of producing LiP, MnP and Laccase and gave much higher degree of lignin degradation in comparison to *P. Chrysosporium* (most studied fungus). It has been reported by Galliano et al. (1991) that in lignin degradation by *Rigidoporous lignosus*, organism does not produce LiP but synthesizes MnP and Laccase. Results also showed that when two enzymes were purified and used in vitro to study their activity, neither enzyme was active independently but when both enzymes were used simultaneously lignin solubilisation was extensive. This shows the synergistic mechanism of their action. Eggert et al. (1996) have reported many natural Laccase mediators such as 3-hydroxy anthranilic acid though experimental data are lacking with respect to their role in lignin biodegradation (Li et al. 2001).

The fungus MVI.2011 used in the present study was found to express all the three extracellular ligninolytic enzymes with appreciable enzymatic activity. However, the exact mechanism of their enzymatic activity has not been understood. In addition to the LiP, MnP and Laccases many other enzymes could be operating in bringing about rapid and almost total lignin utilization.

### Conformation of purified enzyme proteins

**Fig. 2:**
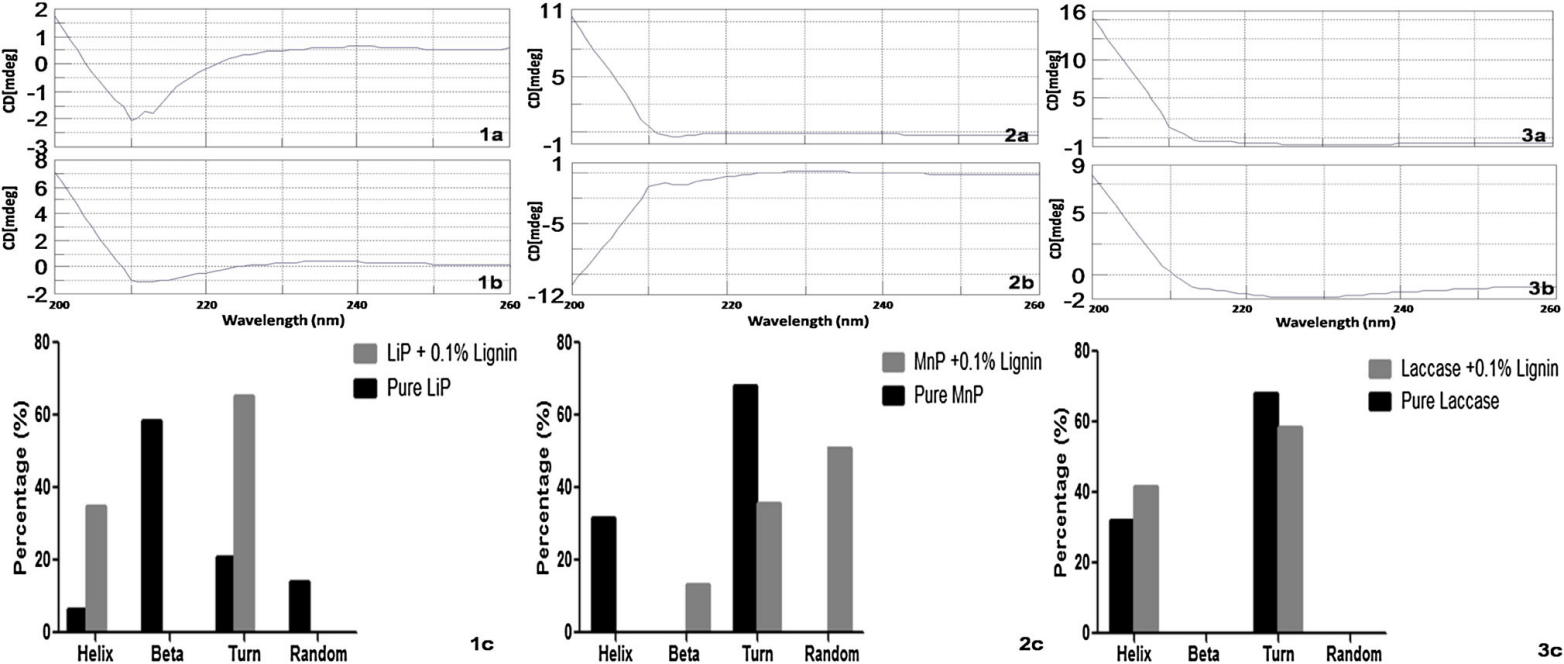
Far UV-CD spectral analysis of pure enzymes: LiP (**1a**), MnP (**2a**) and Laccase (**3a**); pure enzymes in the presence of substrate lignin (**1b**, **2b**, **3b**); and changes in the secondary structure of three enzymes in the presence of lignin (**1c**, **2c**, **3c**)

The circular dichroism spectral study was performed for the three enzymes (LiP, MnP, and Laccase) to obtain some preliminary information on the enzymes protein conformational changes during reaction with substrate. CD spectra suggested that the predominant secondary structure was alpha helix in LiP, and “turns” in MnP and Laccase. CD studies are considered to be important for studying the secondary structure of proteins. The spectral profile of native LiP, MnP and Laccase between 200 and 260 nm recorded in the far-UV (200–260 nm) region is shown in Fig. 2. The dichroic band at 210 nm is seen clearly in all three enzymes characteristic of a-helical structure. As is apparent from the CD spectra the profile of LiP + 0.1 % lignin and Laccase + 0.1 % lignin is somewhat similar to the native enzymes, respectively; however, changes are contrasting in case of MnP + 0.1 % lignin and its native enzyme. Upon interaction with the lignin, a reduction in intensity is seen indicating minor changes contributing to the changes in the protein conformation compared to its free state. In case of LiP, there was almost sixfold (approx. 400 %) increase in the a-helical and threefold increase in “turns” while b-sheets and random structure completely disappeared. In case of MnP, a-helical structure completely disappeared while there was 47.62 % decrease in the “turns”. B-sheets and random structures appeared in the MnP after interaction with lignin. In case of Laccase, there was 29.7 % increase in the a-helical and 14 % decrease in the “turns”, while no b-sheets or random structure were found in Laccase both in its native state and upon interaction. The study of enzymes from MVI.2011 showed that they contained predominantly helix structure. Here the CD data might suggest change in topo chemistry and conformational changes with respect to the affinity of the enzyme for its specific substrate. Lignin is a complex molecule and no optical activity reported in this study (Data not shown). Therefore, it is speculated that lignin though does not have a CD spectra of its own, but on interaction with the enzymes, it does modulate the conformation of three enzymes which might affect its activity. To the best of our knowledge, CD spectral data on purified lignin-degrading enzymes from lignin-degrading microbes before and after complex formation with specific substrates have not been reported before. The changes observed in MVI.2011 LiP, MnP and Laccase enzyme studies can provide a tool to understand the structure and mechanism of action of these enzyme but further studies are required to reach a firm conclusion.

#### Influence of pH, temperature, concentration of substrate and duration of incubation on activity of the purified enzymes

**Fig. 3:**
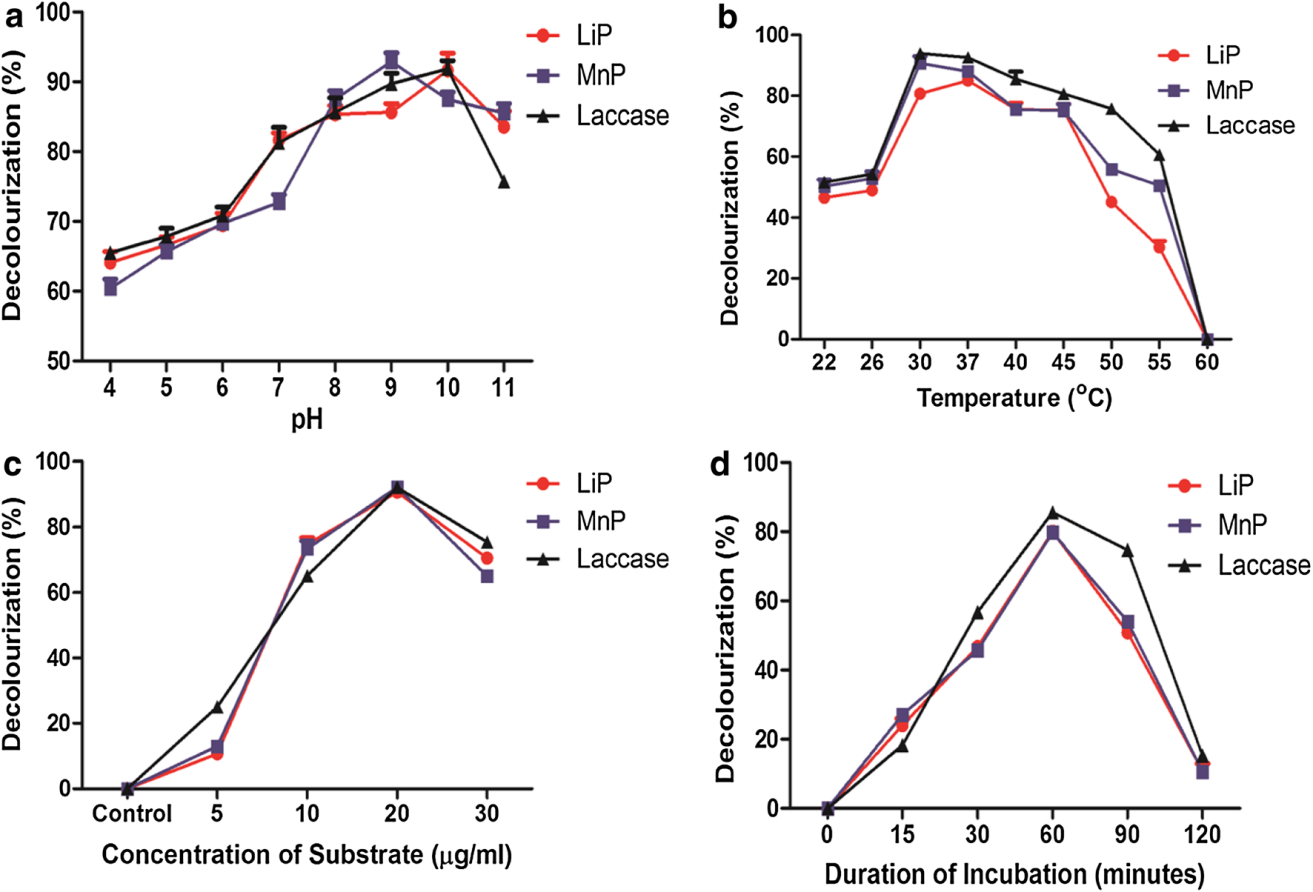
Influence of pH (**a**), temperature (**b**), concentration of substrate (**c**) and duration of incubation (**d**) on pure LiP, MnP and Laccase enzyme. The *error bars* indicate mean ± SD

The ligninolytic enzymes LiP, MnP and Laccase from MVI.2011 were active at pH 4–11. Activity was found to increase with rise in pH of reaction mixture indicated by higher reduction in lignin (Fig. 3a). This observation was remarkable since the organism could grow over a wide range of media with initial pH 4–12. The optimum pH used for culture was pH 9.0. At the end of 18 h, the pH of growth medium fell sharply with concomitant increase in biomass. The culture supernatant or the crude enzyme had a low pH and could be directly assayed for LiP, MnP and laccase at pH 3.5–5.0 as per prescribed standard protocols for ligninolytic enzymes. The purified MVI.2011 enzyme was stable and active at all pH 4–11 with the highest activity at pH 8–11. The LiP, MnP and Laccase enzymes were active at a wide range of temperature from 30 to 55 °C. The highest activity of LiP was observed at 37 °C while MnP and Laccase showed highest activity at 30 °C (Fig. 3b). All three enzyme (LiP, MnP and Laccase) showed highest activity at 20 µg/ml substrate concentration (Fig. 3c). One hour incubation was found to be optimum for all the three enzymes (Fig. 3d).

There are many reports on molecular characterization, influence of pH and temperature on stability and catalytic activity of ligninolytic enzymes, both crude as well as partially purified, from various genera and species of fungi. Regarding the chemical nature it has been proved that LiPs are glycoproteins with an average molecular weight of 38–46 kDa. Detailed studies of enzyme intermediates of LiP and their steady-state and transient state kinetics have shown that these enzymes have their intermediates analogous to the other peroxidases (Renganathan and Gold 1986; Tien et al. 1986; Andrawis et al. 1988; Marquez et al. 1988; Harvey et al. 1989; Wariishi and Gold 1990; Cullen and Kersten 2004). Reports by Edwards et al. 1993 showed that LiP had maximum activity at pH 3.0 and increase in pH caused decrease in activity which has been attributed to the disruption of hydrogen bond formed between heme propionate an aspartic acid residue thereby resulting in complete inactivation of enzyme beyond pH 5. Raghukumar et al. (1999) and Tien and Kirk (1984) on *Flavodon flavus* and *P. chrysosporium*, respectively, have proved the acidic pH optimum of LiP. Report by Ahammed (2002) on the stability study of crude LiP showed that the enzyme was stable for 2 h at pH 2–5 but gradual loss in activity was found with increase in pH to 6 and at higher pH, activity was completely lost. However, contrasting reports (Tuisel et al. 1990) were obtained on LiP stabilities at different pH from *P. chrysosporium* which suggested that though low pH favoured higher activity, stability increased as pH was increased. Ahammed (2002) showed that temperature optimum of LiP was found to be 30 °C and higher temperature beyond 35 °C resulted in the gradual inactivation of LiP. He reported that LiP enzyme activity was retained for a maximum for 2 h over a wide range of temperatures from 20 to 60 °C but higher temperature of 70 °C caused gradual loss in activity. Regarding MVI.2011, though the fungus was isolated from soil from Kerala, India with atmospheric temperatures varying from 28 to 35 °C, the highest growth was found at 37 °C in laboratory-controlled conditions. When the extracellular enzymes are isolated from the genetically controlled regulated cell system and purified, the optima of the pure proteins need not be the same as those for growth with respect to physical properties and/or physiological parameters. This was observed and confirmed by experiments during the initial isolation, screening and preliminary optimization studies (unpublished data, Thankamani 1995). At 25 °C enzymatic activity of separated and partially purified enzyme proteins ranged from 45 to 50 %. Increase in temperature of incubation of enzyme–substrate mixture increased enzyme activity. Assay temperature of 30 °C was chosen as the optimum for all 3 enzymes put together since they demonstrated very marked (80–90 %) lignin degradation at this temperature. Tuisel et al. (1990) had also shown time-dependent loss of LiP activity in *P. Chrysosporium.* At 60 °C the activity was lost completely after 6 h. Farrell et al. (1989) purified LiPs isoenzymes from *Phanerochaete chrysosporium* *BKM*-*1767* and found that they had different iso-electric points between pI 4.7 and 3.3 and molecular weights were found to be between 38 and 43 kDa as determined by SDS-PAGE.

Almansa et al. (2004) showed that Laccase had an average molecular mass of 62 kDa and the iso-electric point to be pH 7 though Laccase had optimum pH of 3.0. Similarly Mabrouk et al. (2010) reported that marine fungal isolate *Trematosphaeria mangrovei* showed higher Laccase activity at pH 4.5 after 14 days of growth while other two ligninolytic activities were not detected. Saito et al. (2003) who isolated and characterized Laccase-producing fungus from soil demonstrated that the iso-electric point (*pI)* of the enzyme was 3.5 and had a molecular mass of approximately 73–80 kDa. The results were similar to the Laccase obtained from Basidiomycete PM1 and white rot fungus *Marasmius quercophilus* which also had an acidic iso-electric point (Dedeyan et al. 2000; Coll et al. 2001). There have been reports by Mishra et al. (2011) who isolated Laccase enzyme from white rot fungal strain (WRF-1) in the economical medium containing synthetic dyes, ground nut shell (GNS) and performed morphological and biochemical analysis. WRF-1 strain was found to decolourize synthetic dyes efficiently at pH 5.0 and 30 °C temperature. The enzyme was purified by chromatography and the molecular mass was found to be 70 kDa by SDS-PAGE. The iso-electric point was found to be 4.8. Spectrophotometer studies showed that the dye was decolorized after 6 days of fermentation. Temperature was considered to be an important factor in controlling composition reaction by affecting microbial metabolism (Tang et al. 2007). Liang et al. (2003) also have reported that increased temperature tends to enhance the microbial activities.

Many classes of effluents discharged from the industries are alkaline in nature owing to the various chemical processes involved. The temperature of the effluents emanating from the industries is also high enough to cause the natural degradation almost impossible. So far, not many reports on biological systems are available which can operate at high pH and temperature of the effluent discharged. So as to carry out the treatment process, the pH has to be reduced considerably either by acid treatment which is more hazardous when discharged into the river or by dilution which adds to its water-intensive nature. The temperature of effluent though gets reduced as it passes through the pipe system and finally discharged, it is still high enough, degradation of which would normally require extra energy to be spent to cool down for normal degradation process. Effluents can be detained in large open tanks for bringing down the temperature before being led to the bioreactor. But cooling to any temperature less than the average ambient temperatures (30–37 °C) prevalent in most parts of India demands lot of electric power; hence, is not a pragmatic or feasible idea as far as industries (distillery, paper pulp, textile, etc.) are concerned. This cost of energy is not viable for the industries, thereby seeking biological system which could act at high temperature and pH and bring about the degradation without involving the costly system is the objective. The study of MVI.2011 culture and purified enzymes obtained from MVI.2011 showed that the optimum enzyme activity of these enzymes was found to be at alkaline pH and at temperature around 30–37 °C which is also the average temperature of most parts of the India. Therefore, it can be used directly for the treatment of paper effluent waste obviating the need for dilution and hence more suitable for continuous operation.

### FTIR

**Fig. 4:**
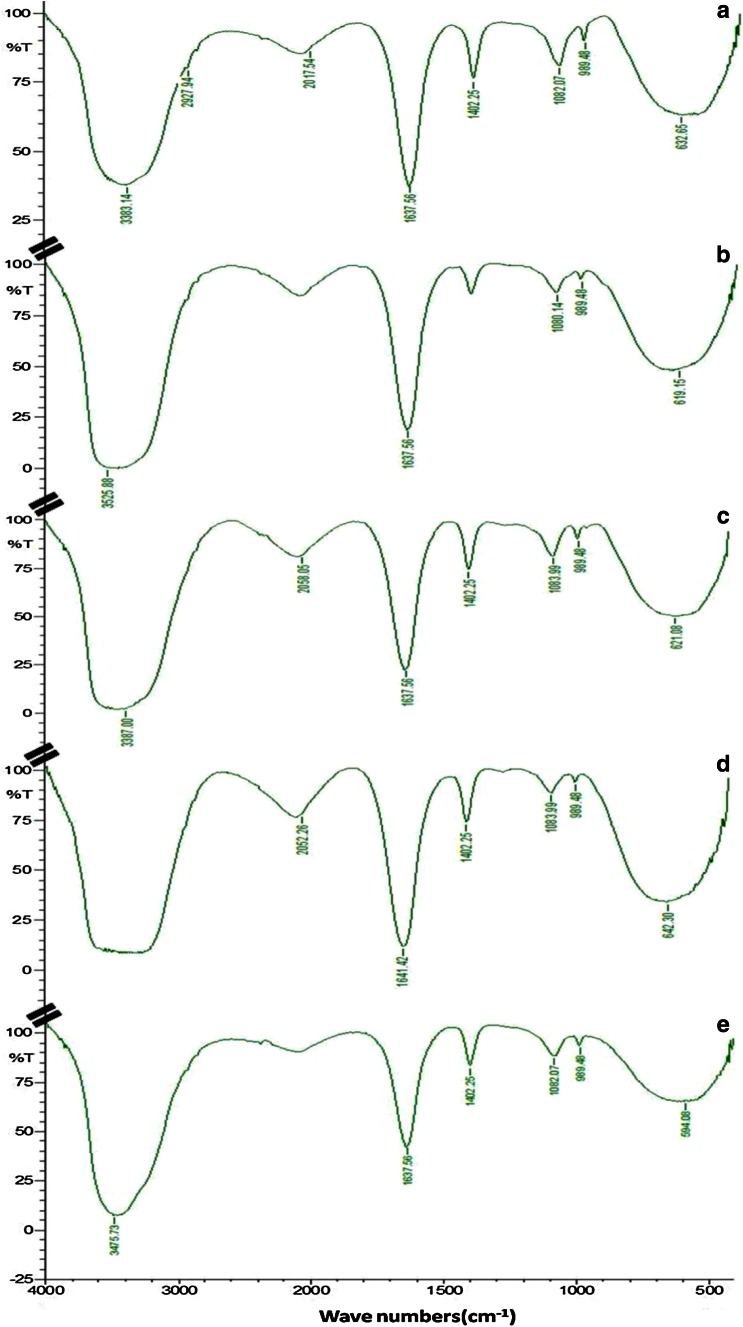
**a** FTIR spectrum of uninoculated minimal mineral media containing 0.1 % commercial lignin. **b** FTIR spectrum of minimal mineral media containing 0.1 % commercial lignin inoculated with MVI.2011. FTIR spectrum of minimal mineral media containing 0.1 % commercial lignin treated with: lignin peroxidase (**c**), manganese peroxidase (**d**) and Laccase (**e**)

Infrared spectra can yield valuable information regarding the chemical groups present in a compound. In the present study, the main effective functional groups on lignin before and after being acted upon by the fungus MVI.2011 have been identified by FTIR (Fig. 4a, b). Main peaks found were broad and strong peak assigned to stretching vibration of aromatic and aliphatic –OH (ν(O–H···O) in lignin at around 3500–3300 cm^−1^, intense peak assigned to ketones (ν(C=O) symmetric stretch) at around 1638 cm^−1^, weak peak assigned to ether (ν(O−C) stretch) at around 1080 cm^−1^. A very weak peak corresponding to stretching vibration of C–H stretch in CH_2_, CH_3_ group of lignin structure was found at around 2930 cm^−1^ which appeared in lignin and disappeared after being acted upon by the MVI.2011. The peaks in the finger print region between 1800 and 600 cm^−1^ were observed and most notably around 1400 cm^−1^ which are often attributed to the lignin components since it arises due to the aromatic skeletal vibration (C=C) in the lignin.

FTIR analysis of lignin degradation with purified enzymes viz. LiP (Fig. 4c), MnP (Fig. 4d) and Laccase (Fig. 4e) showed almost similar peak vibration. Important peaks were assigned to: a very strong and broad peak at around 3500–3300 cm^−1^ (ν(O–H···O) stretching) assigned to stretching vibration of aromatic and aliphatic –OH(alcohol) in lignin, intense peak around 1640 cm^−1^(ν(C=C) symmetric stretch) assigned to ketones and medium intensity peak ascribed to aromatic stretch (ν(C–C) conjugated with C=C) at 1402 cm^−1^ and peaks of variable intensity were found around 1080 cm^−1^ assigned to ether (ν(O–C) stretch). All three enzymes showed almost similar pattern of peak with some differences in the peak intensity which might suggest that lignin degradation pathway by three enzymes shares similar breakdown pathway. The peak at around 1402 cm^−1^ remained almost constant upon degradation by the three enzymes; however, the intensity reduced when the lignin breakdown occurred by MVI.2011 showed that these three enzymes might not be responsible for breakage of aromatic ring. The variable intensity of the peak at around 1080 cm^−1^ means that –O– asymmetric stretching vibration in the ether bond and the C–O stretching vibration also showed some variation upon being acted by the MVI.2011 and three purified enzymes. This might be due to the generation of more easily degradable materials after the oxidation and demethylation of lignin side chain (Liu et al. 2014).

Detailed study and analysis of culture supernatants at very close time intervals need to be carried out to understand and affirm the sequential appearance and disappearance of functional groups to identify formation of intermediates and end products of metabolic breakdown of lignin by MVI.2011. It may be surmised that in a system where whole cells are used or in a natural process, in addition to the three enzymes studied some other enzymes secreted by the microorganism could work synergistically by oxidative pathways to mineralise lignin completely into carbon dioxide and water under optimum conditions. Careful analysis of the spectral peaks showed that though similar functional groups appeared both in MVI.2011 culture broth and in purified isolated MVI enzyme-treated lignin a mild shift in the peaks was observed. The identification of the functional groups in the lignin-degraded products corresponded to the breakdown products identified by the mass spectrometry. In a similar finding by Yang et al. (2010), the white rot fungi generated a great amount of conjugated and unconjugated C=O which may be due to oxidative mechanism involved in lignin biodegradation.

### GC–MS studies

**Fig. 5:**
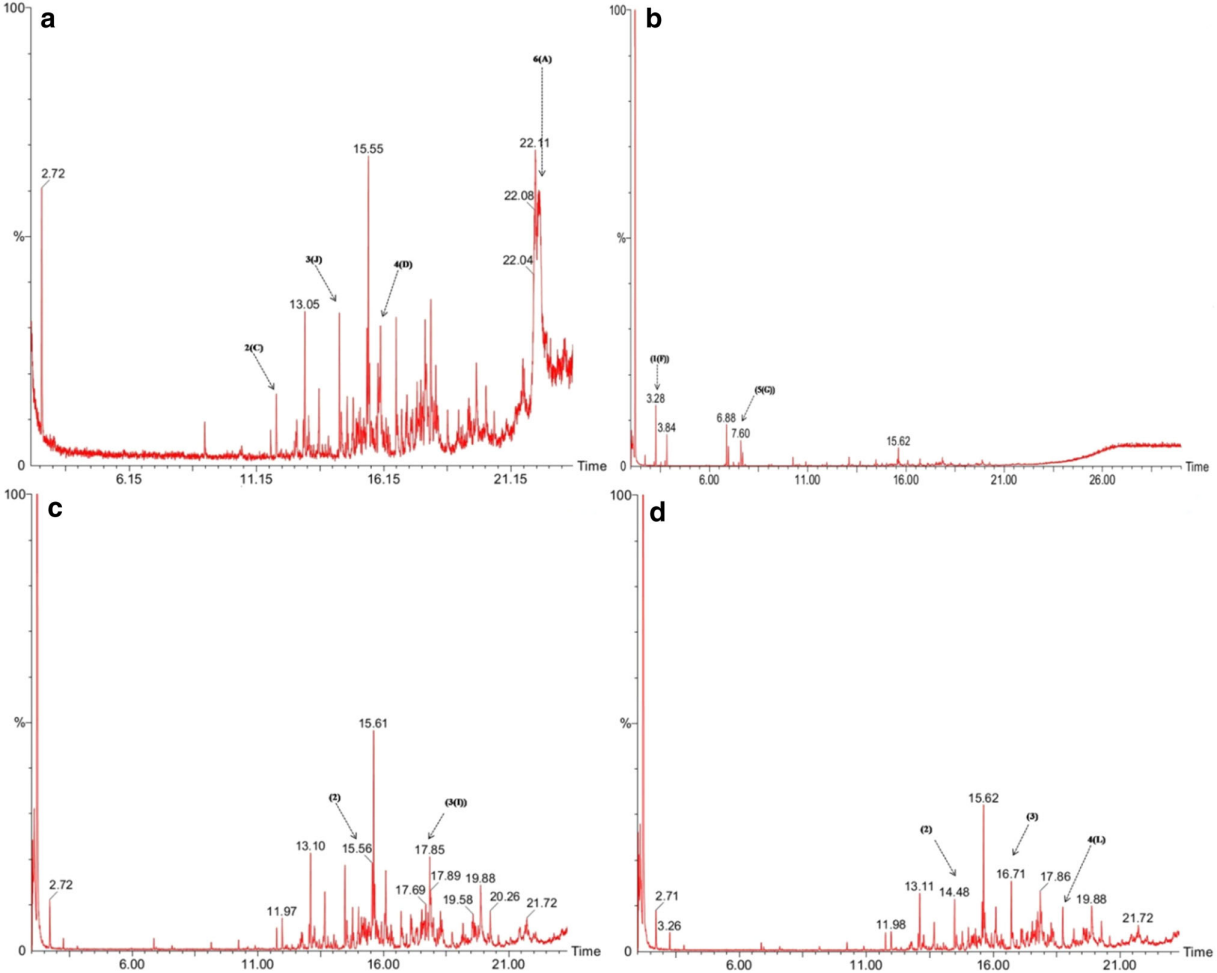
GC chromatogram of minimal mineral media containing 0.1 % commercial lignin treated with: **a** MVI.2011 **b** Lignin peroxidase (LiP), **c** manganese peroxidase (MnP), **d** Laccase. *Number in the bracket* corresponds to the identifier described in Table 2. Control containing only lignin could not be identified in the GC

The breakdown products released from the lignin degradation by three purified enzymes (LiP, MnP, Laccase) produced by MVI.2011 were analysed by GC–MS. The total ion chromatograph (TIC) patterns corresponding to the degradation pattern of compounds are shown in Fig. 5 and their peak identity and mass spectral data are shown in Table 2. The control (data not shown) did not show any peak as the intact lignin molecule was undetectable by the GC–MS.

**Table 2:** Identification of chromatographic peak of breakdown products from lignin degradation

No. (identifier)	RT (GC)	*m*/*z* of major peaks (abundance)	Identified/suggested compound	Potential application
MVI.2011				
1 (B)	9.12	57 (100), 71 (50), 85 (18), 98 (4), 173 (4), 218 (6), 266 (5), 327 (6)	Tridecane, 2,2,4,10,12,12-hexamethyl-7-(3,5,5-trimethylhexyl)	Component of biofuel
2 (C)	11.93	57 (100), 71 (90), 85 (38), 99 (5), 155 (4), 216 (3), 323 (4), 570 (6)	Hentriacontane	NI
3 (J)	14.41	57 (100), 71 (57), 85 (48), 99 (6), 115 (3), 155 (2)	Dodecane,1-fluoro	Component of biofuel
4 (D)	16.03	55 (36), 69 (42), 71 (100), 85 (99), 113 (17), 127 (9), 168 (5)	Pentane, 3-(Bromomethyl)	NI
5 (E)	18.05	55 (21), 68 (18), 70 (100), 71 (9), 86 (31), 96 (8), 154 (22)	Endo-3-acetamidocamphor	NI
6 (A)	22.12	58 (100), 70 (55), 84 (72), 91 (54), 125 (68), 153 (22), 197 (13), 207 (36), 281 (9)	1-Propanamine, *n*,*n*-dimethyl-3-[[1-(phenylmethyl)-1 h-indazol-3-yl]oxy]	NI
LiP				
1 (F)	3.28	57 (68), 85 (100), 86 (8), 112 (3), 128 (5)	Oxalic acid, 6-ethyloct-3-yl isobutyl ester	Dyeing industry, mordant
2	6.87	57 (100), 71 (88), 85 (59), 99 (7), 113 (14), 127 (9), 170 (3), 259 (2)	Hentriacontane	NI
3	6.97	57 (100), 71 (72), 85 (64), 113 (7), 126 (3), 165 (2)	Dodecane,1-fluoro	Component of biofuel
4 (H)	7.22	57 (60), 69 (100), 83 (38), 99 (5), 111 (9), 125 (4), 167 (6)	1R,2C,3T,4T-Tetramethyl-Cyclohexane	Solvent
5 (G)	7.60	55 (32), 71 (100), 85 (48), 113 (9), 127 (10)	1-Hexanol, 5-methyl-2-(1-methylethyl)	Solvent
MnP				
1 (K)	11.76	55 (17), 77 (16), 93 (46), 105 (90), 119 (100), 133 (7), 161 (72), 204 (11)	Alpha cubebene	Neuroprotective
2	15.56	57 (100), 71 (63), 85 (66), 99 (18), 113 (22), 127 (8), 169 (6), 236 (3), 374 (4)	Hentriacontane	NI
3 (I)	17.85	69 (37), 71 (100), 85 (49), 99 (29), 113 (21), 127 (17), 155 (8), 169 (6), 183 (4), 266 (3)	Octadecane, 2,6,10,14-tetramethyl	NI
Laccase				
1	11.76	77 (22), 93 (60), 105 (88), 119 (100), 161 (82), 204 (5)	Alpha.cubebene	Neuroprotective
2	14.48	55 (50), 71 (100), 85 (21), 97 (10), 113 (7), 226 (6)	Dodecane, 1-fluoro	Component of biofuel
3	16.71	57 (100), 71 (56), 85 (32), 99 (10), 113 (8), 127 (6), 254 (4), 386 (3)	Hentriacontane	NI
4 (L)	18.75	57 (100), 71 (48), 85 (42), 99 (14), 113 (6), 127 (4), 174 (3)	Nonane, 2,2,4,4,6,8,8-heptamethyl	Component of biofuel

**Fig. 6:**
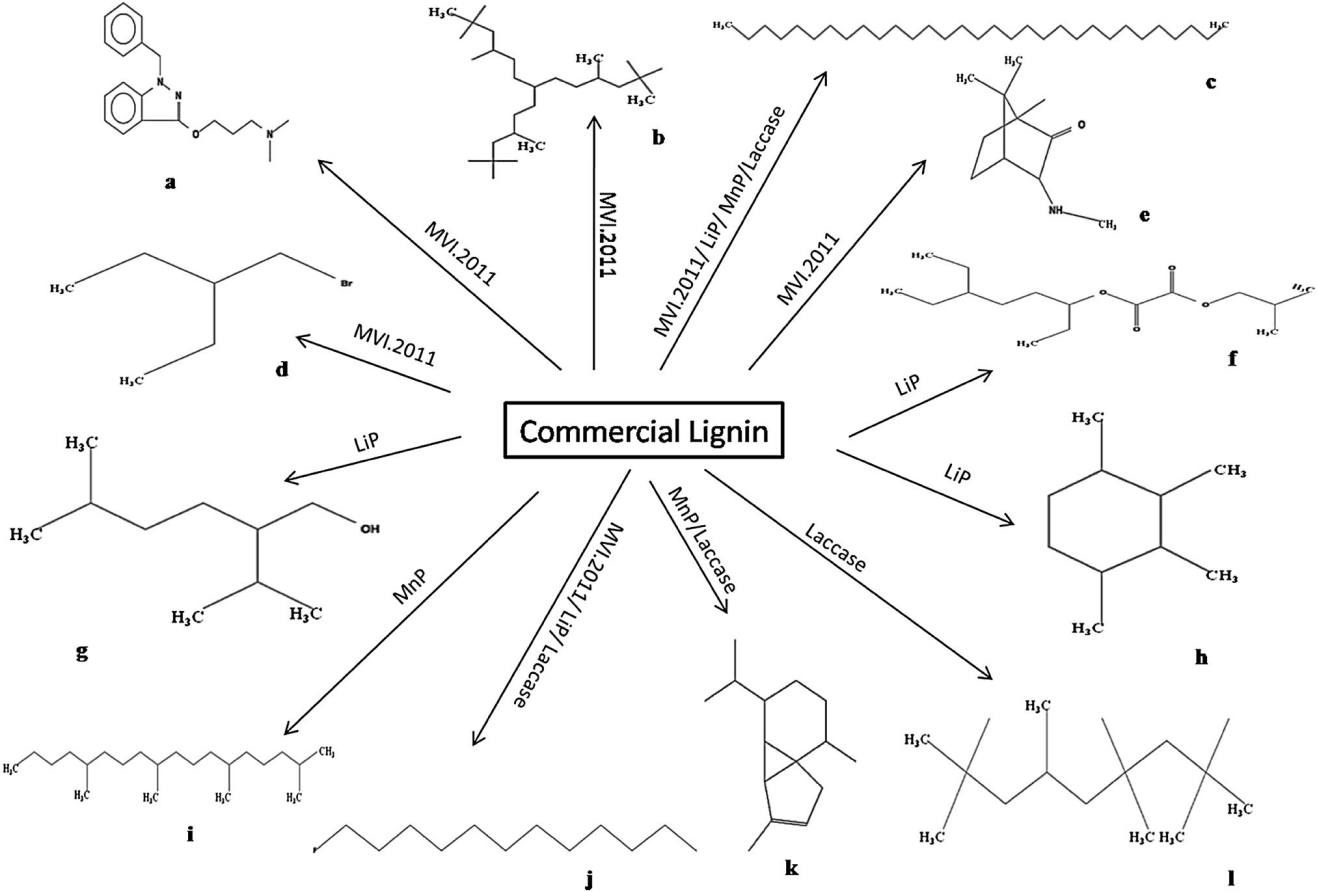
Complex of lignin breakdown products by MVI.2011 and three purified enzymes: LiP, MnP and Laccase as identified in the GC–MS. Identification of chemical compounds has been described in Table 2 (*first column with alphabet* [*a*–*l*])

Identifier in bracket corresponds to the chemical structure in Fig. 6

*NI* not identified

The number of peaks in the TIC pattern increased significantly after being treated with MVI.2011 and three purified enzymes compared to control. Many low molecular compounds, which could not be identified in the control, were identified following degradation of lignin by the three MVI.2011 enzymes. They were oxalic acid, 6-ethyloct-3-yl isobutyl ester; hentriacontane; 1-hexanol, 5-methyl-2-(1-methylethyl); 1R,2C,3T,4T-tetramethyl-cyclohexane; alpha cubebene; octadecane, 2,6,10,14-tetramethyl and nonane, 2,2,4,4,6,8,8-heptamethyl. The compounds like oxalic acid derivative compounds identified in this study are guaiacol related which might be produced due to the oxidation of guaiacyl units present in the precursor coniferyl alcohol. These precursors along with syringyl units from precursor sinapyl alcohol form the basic moieties that are essential components of lignin structure (Shi et al. 2013). A report by Kingsley et al. (2002) showed organic acid production by fungus *Phanerochaete chrysosporium* in which important organic acids produced were malonic acid and oxalic acid as shown in the present study. However, present study did not clearly reveal any syringyl-related compounds. Aromatics compounds and some aldehyde-ketone type compounds were identified due to degradation of lignin and this is well in accordance with the FTIR result. The identification of low molecular weight compounds in the extract of MVI.2011 culture and ones treated with three enzymes favours the conclusion that lignin was efficiently degraded by this novel organism. The complex of lignin breakdown product is illustrated in Fig. 6. It is noteworthy that most of the compounds produced can find suitable application in the alternative industry and most importantly as an important product for use as a biofuel. Oxalic acid derivative is an important component of dyeing industry: used as mordant and also used as a component in baking powder (Riemenschneider and Tanifuji 2011). Cubebene and its iso-forms have been used as an important component of herbal formulations and have recently drawn attention of the researchers for their neuron protective effect (Park et al. 2013, 2015). However, how far these compounds produced by lignin degradation using this novel organism can find application as an alternative medicine or in other industries is yet another area worth exploring. Higher alkanes like octadecane, nonane produced from lignin as by-products upon degradation are some of the most important components of fuel oil and lubricating oil (Wackett 2008). Fungal breakdown of lignin by MVI.2011 into such alkanes could be part of humus in the natural degradation occurring in the soil ecosystem. A class of degradation products of recalcitrant dyes by white rot fungus was recently reported by Mishra et al. (2011) and some findings about the degradation products were also reported by Telke et al. (2010) in case of *Aspergillus ochraceus* NCIM-1146. The degradation products find important application in industries and specific products like dodecane has recently generated a considerable amount of attention as a potential surrogate fuel for application in Jet fuels. The application potential of lignin bio-products in several industries like food and flavour industry and chemical industries can be enhanced if the bio-catalytic breakdown of lignin could be harnessed and controlled. A more protracted in-depth qualitative analysis of the innumerable products in the MVI 0.2011 culture supernatant might reveal presence of valuable lignin by-products which have application in the food industry. These data illustrate the potential of novel fungus MVI.2011 in conversion of lignin to value-added chemicals; however, large-scale production of such value-added products continues to be a challenge.

Regarding the mechanism of lignin degradation, it is reported that hydrogen peroxide is an important requirement for oxidation by LiP and MnP enzymes. In case of several white rot fungi including *P. chrysosporium,* this requirement is met by extracellular oxidases that reduce molecular oxygen into H_2_O_2_ and thereby oxidizing the co-substrate. One such extracellular enzyme involved in peroxide production is glyoxal oxidase (GLOX) (Kersten 1990). According to the unpublished data (by V Thankamani and TR Sreekrishnan), it was shown that fungus MVI.2011 released H_2_O_2_ during lignin breakdown. There is another route called Aryl alcohol oxidases (AAOs) which has been studied in some white rot fungi for H_2_O_2_ production. In some species of *Bjerkandera*, chlorinated anisyl alcohols are reduced by AAO to produce H_2_O_2_ (De Jong et al. 1994).

## Conclusion

We aimed to illustrate for the first time the decay mechanism of lignin by the ligninolytic enzymes secreted by MVI.2011, a “novel” fungus. Though the available literature abounds in data on lignin-degrading enzymes from different fungi, the “novel” fungus MVI.2011 has been found to secrete enzymes which serve to completely decompose and mineralise lignin over a wide range of pH and temperature, conditions under which generally fungi and other available biological alternatives fail to perform efficiently. Therefore, it would be worthwhile to try to explore such biological entities further for various biotechnological applications including degradation of dyes or dye-based effluent, tannery waste, aromatic hydrocarbons which form important part of lignin structure and pre-treatment of recalcitrant natural lignocelluloses biomass for use as biofuels (Joshi and Gold 1993; Wolter et al. 1997; Hofrichter et al. 1998; McMullan et al. 2001; Stolz 2001). It can be concluded from the spectroscopic data (FTIR and GC–MS) that lignin both purified and natural when acted upon by the fungus MVI.2011 was broken down by enzymes LiP, MnP and Laccase along with several others to a considerable extent including opening up of aromatic rings and c–c bonds, formation of organic acids, higher alkanes, etc. So the direct evidence for structure alterations of lignin during MVI.2011 degradation from the present study provides a useful idea about the industrial and commercial application of the organism especially in pulp and paper industry.

## Electronic supplementary material

Below is the link to the electronic supplementary material.

Supplementary material 1 (DOCX 236 kb)
