# Supplementary material for: Characterization of lignin-degrading enzymes (LDEs) from a dimorphic novel fungus and identification of products of enzymatic breakdown of lignin
Source: 3 Biotech. 2016 Feb 13;6(1):56. doi: 10.1007/s13205-016-0384-z (PMC4752945; doi:10.1007/s13205-016-0384-z)
Supplement: Supplementary file 1 — Supplementary material 1 (DOCX 236 kb) [file 13205_2016_384_MOESM1_ESM.docx]

**List of Supplementary Figure:**

**Supplementary Figure 1: HPLC graphs showing single peaks in the GPC fraction purified enzymes LiP (a), MnP (b), Laccase (c)**

**
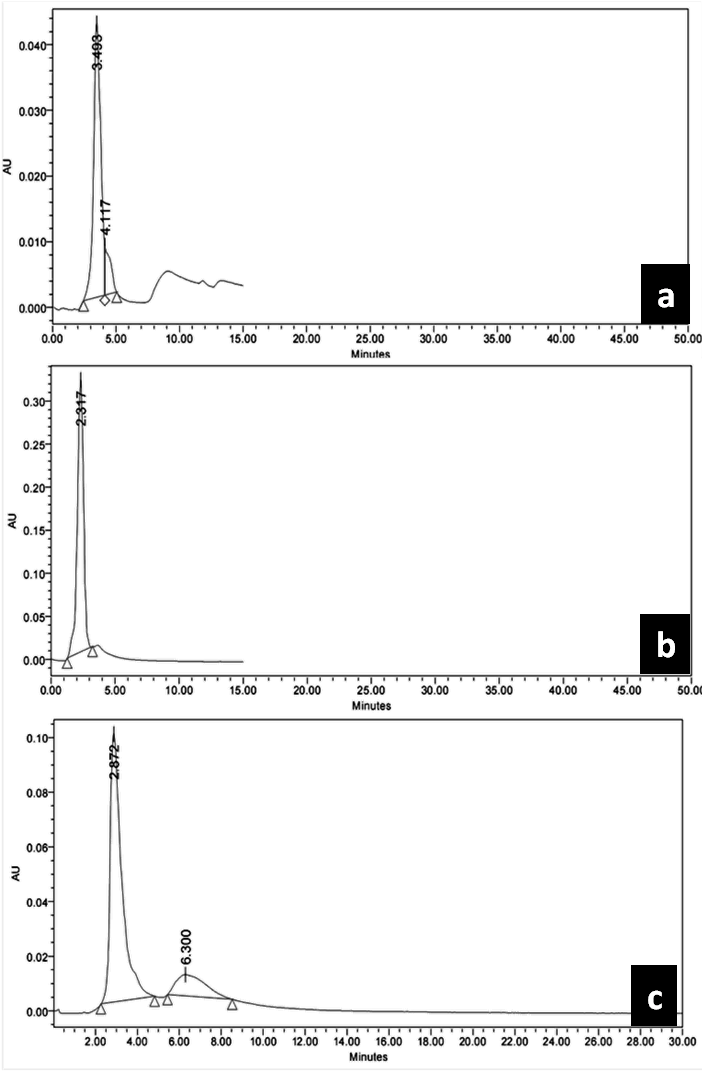
**
